# The chemical theory of valence

**DOI:** 10.1107/S2052520625000022

**Published:** 2025-02-12

**Authors:** Ian David Brown

**Affiliations:** a Brockhouse Institute for Materials Research McMaster University Hamilton OntarioL8S 4M1 Canada; Georgetown University, USA

**Keywords:** chemical theory of valence, structure, bonding

## Abstract

Relaxing the restriction that valence can only adopt integer values removes the distinction between ionic and covalent bonds, resulting in a simple and powerful valence theory using the concept of the bonding strength of an element, defined as the ratio of its atomic valence to its average coordination number. Bonding strength, closely related to electronegativity, determines which atoms form stable bonds and the chemical properties of the resulting compounds of both organic and inorganic compounds.

## Abstract 2

Relaxing the restriction that valence can only adopt integer values removes the distinction between ionic and covalent bonds, resulting in a simple and powerful valence theory that is able to predict the structures and chemical properties of both organic and inorganic compounds.

## 1. Introduction

The oldest theory of chemical structure and bonding is the valence theory that was developed in the 1860s to explain the structures of organic molecules. It continues to be used by organic chemists today because it provides a simple picture of atoms and bonds that complements the more powerful quantum description. However, attempts to extend the valence theory to inorganic chemistry have all failed because of the mistaken belief that the valence of an atom consists of discrete electrons and therefore can only adopt integral values (Lewis, 1923 ▸). Remove that restriction, and a rich and predictive valence theory appears, one that can describe all types of localized bonding, both organic and inorganic.

## 2. Valence defined

Valence is not restricted to integral values, because discrete electrons cannot be identified in atoms or molecules. When an electron has been captured by an atom it is absorbed into the continuum of negative charge that surrounds the nucleus. The valences shown in the periodic table are restricted to integers because the process of ionization can only add or remove charge from an atom one electron at a time. Once the electron has been absorbed into the atom, this restriction no longer applies.

The valence of an atom is the amount of negative charge the atom has available for bonding, with one unit of valence charge being equal to one unit of electron charge. A chemical bond is formed when the valence charges of two atoms overlap. There is no requirement that the charge forming the bond be restricted to integral values. Relaxing the integer constraint allows valence theory to describe the structures and reactions of complex inorganic materials and aqueous solutions, as well as both the inter- and intra-molecular bonding in organic compounds (Brown, 2023 ▸).

## 3. The role of geometry

Chemical structures can be described using either Euclidian or coordinate geometry. Quantum mechanics, being a force field theory, requires the use of coordinate geometry, because it needs the three-dimensional spatial coordinates of the nuclei in order to calculate the fields and energies. Coordinate geometry describes a particular arrangement of the nuclei, and any change in the positions of these nuclei results in a change in the energy. Valence theory uses Euclidian geometry, a graphical theory with no knowledge of spatial coordinates. A chemical structure is displayed as an array of atoms linked by bonds, where each bond describes the chemical, rather than the physical quantum interaction between a pair of atoms. The atoms are not pinned to particular locations, hence valence theory cannot be used to describe force fields or to calculate energies. Instead it uses a free-floating network that makes it easy to explore a variety of arrangements and deduce the chemical properties of each.

## 4. The chemical properties of atoms

In the chemical valence theory atoms are characterized by just two chemical properties: their valence and their size, and in any stable network of bonds these properties must obey just three simple rules. According to the IUPAC definition (McNaught & Wilkinson, 1997 ▸), the *valence*, *V*, of an atom is determined from observed chemical compositions, and in a chemical context its size is conveniently described by its typical coordination number, *i.e.* the number of neighbours to which it can bond: the larger the atom the more neighbours it can accommodate. Although a given element may adopt a range of coordination numbers, these numbers tend to cluster around an average that can then be taken as the atom’s *typical coordination number*, *N* (Gagné & Hawthorne, 2016 ▸; Gagné & Hawthorne, 2018*a* ▸; Gagné & Hawthorne, 2018*b* ▸). The ratio of these two numbers, *V*/*N* = *S*, is the *bonding strength* of the atom (Gagné & Hawthorne, 2017 ▸). This is the most important chemical property of an atom because it is the amount of valence that the atom uses to form a typical bond. Stable bonds can only be formed between atoms with similar bonding strengths. There is no necessary requirement in this theory for *V*, *N* or *S* to be an integer.

## 5. The rules for forming bond networks

In the chemical valence theory, a stable network of bonds obeys three rules: (1) each atom distributes its valence among the bonds that it forms, (2) two atoms form a bond by contributing equal amounts of valence. This amount, known as the *bond valence*, serves as a characteristic measure of the strength of the bond, (3) consistent with the first two rules, equilibrium is achieved when the difference between the bond valences and the average bond valence around each atom is minimized.

### 5.1. An example

Divalent oxygen with *V* = 2 valence units (vu) and *N* = 4 has a bonding strength *S* = 0.50 vu. It is observed that under normal conditions, two atoms will only form bonds if their bonding strengths differ by less than a factor of two. Oxygen is therefore expected to form stable bonds with atoms whose bonding strengths lie in the range between 0.25 to 1.00 vu. The bonding strength of sodium (*S* = 0.17 vu) is too small to bond to oxygen, thus Na_2_O is unstable and readily reacts with H_2_O and CO_2_ in the air to form hydrogen carbonates with a better valence match, but magnesium (*S* = 0.33 vu) forms the stable oxide MgO. The match between Mg and O is not perfect but by adopting a coordination number of six, oxygen can form bonds with a valence of 0.33 vu to match the bonding strength of magnesium. The special conditions that allow oxygen to form bonds stronger than 1.00 vu are discussed below.

## 6. The picture of an atom in valence theory

As noted above, valence theory uses only two properties to fully characterize an atom: its valence and its typical coordination number, but sometimes a physical picture of the atom helps us to understand how these properties arise and how they are best applied. A physical picture also allows the atom of the valence theory to be compared to the physical picture of an atom provided by quantum mechanics. In valence theory the physical atom is simple; it is defined as being identical to the isolated free atom under all circumstances, whether it is free or whether it is part of a molecule or crystal. Atoms in valence theory are therefore always spherically symmetric and always electroneutral, ions are never used and atoms are never charged. This picture may appear to be an oversimplification of the physics, but when these atoms are placed at their observed positions in a molecule, the resulting charge density differs from the observed charge density by less than 1.0 e Å^−3^, a difference that is fully explained by the Pauli exclusion principle. In valence theory the atom can be divided conceptually into a positively charged spherical core surrounded by a negatively charged spherical valence shell. This division of the atom is conceptual rather than physical because the physical locations of the core and valence shell cannot in principle be identified. A bond is formed in this conceptual space by the overlapping of portions of the valence shells of two neighbouring atoms. The charge in the overlap region belongs to both atoms, and as such it attracts both cores, binding them together to form the bond. In the valence theory charge is never transferred from one atom to the other, it always remains with its own atom, thereby greatly simplifying the theory (Raebiger *et al.*, 2008 ▸). There is no need to keep track of ions or charge transfer, they play no role in valence theory.

## 7. Bond valences and bond lengths

There is a good empirical correlation between the valence of a bond and its length: the larger the valence the shorter the bond (Gagné & Hawthorne, 2015 ▸). This correlation allows observed bond lengths to be converted to bond valences, or alternatively, bond lengths can be predicted from calculated bond valences. Small differences between the predicted and observed bond lengths provide information about constraints that are not part of valence theory. Some arise from the distortions that stabilize degenerate quantum states in transition metals (Dunitz & Orgel, 1960 ▸), but most are strains caused by contacts between non-bonded atoms. They provide information about stresses that are responsible for potentially important properties, such as phase transitions and hydrogen bonding as described below (Lufaso & Woodward, 2001 ▸).

## 8. Electronegativity determines bond polarity and drives the adoption of lower atomic valences

The Pauling electronegativity series closely follows the series of bonding strengths calculated using the element’s largest allowed valence (Brown & Skowron, 1990 ▸). This equivalence is not surprising. If one assumes that the typical coordination number, *N*, is proportional to the surface area of the atom, the bonding strength, namely *V*/*N*, is proportional to the electric field of the valence charge at the surface of the atom (Allred & Rochow, 1958 ▸). In cases where bond polarity is important, the atom with the smaller electronegativity is defined as the Lewis base (*i.e.* cation) and the atom with the larger electronegativity is the Lewis acid (*i.e.* anion). Bond valences are treated as positive at the Lewis base and negative at the Lewis acid, a sign convention that reflects the relative charge densities in the valence shell. In all these cases the atoms themselves remain electrically neutral. The electronegativity also determines which of the allowed valence states an atom will adopt. Where the difference in the electronegativities of two bonded atoms is greater than 0.5 vu, the Lewis base uses only a part of its valence shell for bonding, thereby lowering its atomic valence. For example, the difference in the electronegativities of magnesium and oxygen is 1.67 vu, but a stable bond can be formed if oxygen uses only two of its valence shell units for bonding. The remaining four units become *non-bonding valence*, traditionally known as lone electron pairs. The ability of atoms with large electronegativities to adopt lower atomic valences greatly increases the range of compounds that they can form (Gillespie & Hargittai, 1991 ▸). Although both the electronegativity and the bonding strength are defined as the ratio of valence to coordination number, the term ‘bonding strength’ uses only the experimentally observed values of *V* and *N* as described above. The electronegativity is calculated using all the valence charge in the valence shell.

## 9. Non-bonding valence (lone pairs)

The valence shell of divalent oxygen contains 2.00 vu of bonding valence and 4.00 vu of non-bonding valence. In MgO, where the bonding strength of magnesium (0.33 vu) is less than that of oxygen (0.50 vu) the bonding and non-bonding valences are both distributed uniformly around the spherical valence shell of oxygen, resulting in oxygen forming six symmetrically arranged bonds each having a bond valence of 0.33 vu. However, when oxygen bonds to an atom with a bonding strength greater than 0.50 vu, oxygen can increase its bonding strength to match that of the Lewis acid by increasing the amount bonding valence in the bond overlap region (Alcock, 1972 ▸). In order to maintain the spherical symmetry of the valence shell, some of the non-bonding valence must be moved out. In this case the non-bonding valence (*i.e.* lone pairs) are said to be stereoactive. For example, the bond strength of aluminium (0.57 vu) is greater than that of oxygen (0.50 vu). Oxygen moves an extra 0.07 vu of bonding valence into the region of the Al—O bond, forcing an equal amount of non-bonding valence to become stereoactive. This results in the oxygen atoms in corundum (Al_2_O_3_) forming two strong bonds of 0.57 vu to match the bonding strength of aluminium, and two weaker bonds of 0.43 vu around the partially stereoactive lone pairs. In extreme cases, the non-bonding valence can become fully stereoactive, as found in CO_2_, where each oxygen atom uses all of its 2.00 vu of bonding valence to form a C—O bond, leaving the opposite side fully occupied by non-bonding valence (lone pairs). Intermediate degrees of stereoactivity are found in the series of *X*O_4_ complexes (*X* = P, S, Cl), where the valences of the *X*—O bonds match the bonding strengths of the *X* atoms: 1.25, 1.50 and 1.75 vu, respectively (Bickmore *et al.*, 2013 ▸). The bonding valence of oxygen that is not used to form the *X*—O bond remains on the outside of the *X*O_4_ complex where it is used to form an external bond to weaker Lewis bases.

## 10. Non-bonding contacts: the hydrogen bond and aqueous chemistry

The hydrogen bond illustrates the chemistry that results when the cores of two non-bonded atoms are forced into close contact. When hydrogen (valence of 1.00 vu) forms bonds to two oxygen atoms, the expectation from rule 3 is that both O—H bonds will have a valence of 0.50 vu, but such a symmetric arrangement would force the cores of the two oxygen atoms to overlap. This compressive stress is relaxed by moving the hydrogen atom closer to one oxygen, weakening the other O—H bond, causing it to become longer, until the two oxygen cores are just touching (Brown, 1995 ▸). An analysis of coordination numbers and non-bonding O⋯O distances shows that the repulsive stress is relaxed when the O—H and H⋯O bonds have valences of 0.81 and 0.19 vu, respectively. This asymmetry is responsible for the existence of molecular water (H_2_O) in which both the hydrogen and oxygen atoms have external bonding strengths of 0.19 vu, allowing water, according to rule 2, to bond to both Lewis acids and Lewis bases with bonding strengths ranging from 0.09 to 0.38 vu, a range that includes a large number of elements and complexes, including water itself. Water is thus an ideal solvent for atoms that form weak bonds. At equilibrium all the species present in an aqueous solution must have the same bonding strength. This is achieved by attaching or detaching hydrogen atoms, with consequent predictable changes in the pH. Valence theory also provides a more nuanced picture of the structures and reactivities of the organic compounds that form hydrogen bonds.

## 11. Final thoughts

The formal development of the theory outlined here, together with references, can be found in Brown (2023 ▸), which shows that valence is the property that most naturally describes the chemical, as opposed to the physical, interactions between atoms. Although the concepts of bond and valence are central to chemical discourse, they are concepts that have proved surprisingly difficult to define. They do not arise naturally from quantum mechanics, but in the 19th century they did bring order to the complexities of organic chemistry. The ill-conceived concepts of ionic and covalent bonds were a necessary consequence of the later association of valence with the indivisible electron, but this association, while it appeared to make sense at the time, has only led to confusion. The ionic–covalent model offers little chemical insight and makes few predictions. Removing the electron, a quantum concept that has no place in valence theory, allows ‘valence’ and ‘bond’ to speak with their own natural voice. The result is a picture that gives a simpler, yet more complete account of the chemical interactions between atoms. For many people this may be all chemical theory of bonding they need, but for those who later wish to engage with the complexities of quantum mechanics, it provides a simple complementary picture expressed in the language of chemistry.
